# Super-Resolution Ultrasound Imaging Provides Quantification of the Renal Cortical and Medullary Vasculature in Obese Zucker Rats: A Pilot Study

**DOI:** 10.3390/diagnostics12071626

**Published:** 2022-07-04

**Authors:** Stinne Byrholdt Søgaard, Sofie Bech Andersen, Iman Taghavi, Carlos Armando Villagómez Hoyos, Christina Christoffersen, Kristoffer Lindskov Hansen, Jørgen Arendt Jensen, Michael Bachmann Nielsen, Charlotte Mehlin Sørensen

**Affiliations:** 1 Department of Diagnostic Radiology, Rigshospitalet, 2100 Copenhagen, Denmark; posttilsofiebech@gmail.com (S.B.A.); lindskov@gmail.com (K.L.H.); mbn@dadlnet.dk (M.B.N.); 2 Department of Biomedical Sciences, University of Copenhagen, 2200 Copenhagen, Denmark; christina.christoffersen@regionh.dk (C.C.); cmehlin@sund.ku.dk (C.M.S.); 3 Department of Clinical Medicine, University of Copenhagen, 2200 Copenhagen, Denmark; 4 Center for Fast Ultrasound Imaging, Department of Health Technology, Technical University of Denmark, 2800 Lyngby, Denmark; imat@dtu.dk (I.T.); jaje@dtu.dk (J.A.J.); 5 BK Medical ApS, 2730 Herlev, Denmark; carlos.villagomezhoyos@gmail.com; 6 Department of Clinical Biochemistry, Rigshospitalet, 2100 Copenhagen, Denmark

**Keywords:** ultrasound localization microscopy, image analysis, chronic kidney disease, metabolic syndrome, nephropathy, obesity

## Abstract

Obesity is a risk factor of chronic kidney disease (CKD), leading to alterations in the renal vascular structure. This study tested if renal vascular density and tortuosity was quantifiable in vivo in obese rats using microbubble-based super-resolution ultrasound imaging. The kidneys of two 11-week-old and two 20-week-old male obese Zucker rats were compared with age-matched male lean Zucker rats. The super-resolution ultrasound images were manually divided into inner medulla, outer medulla, and cortex, and each area was subdivided into arteries and veins. We quantified vascular density and tortuosity, number of detected microbubbles, and generated tracks. For comparison, we assessed glomerular filtration rate, albumin/creatinine ratio, and renal histology to evaluate CKD. The number of detected microbubbles and generated tracks varied between animals and significantly affected quantification of vessel density. In areas with a comparable number of tracks, density increased in the obese animals, concomitant with a decrease in glomerular filtration rate and an increase in albumin/creatinine ratio, but without any pathology in the histological staining. The results indicate that super-resolution ultrasound imaging can be used to quantify structural alterations in the renal vasculature. Techniques to generate more comparable number of microbubble tracks and confirmation of the findings in larger-scale studies are needed.

## 1. Introduction

The global obesity pandemic affects more than 650 million adults [1] and has a huge impact on worldwide socioeconomics, as well as medical consequences. Overweight and obesity are important pathogenic factors in ~14–30% of patients with chronic kidney disease (CKD) [2,3]. Being able to identify obese individuals with increased risk of CKD is crucial to preventing the development of the disease. Obesity is often associated with hypertension and diabetes: the two main factors in promoting renal dysfunction. Increased generation of inflammatory cytokines from adipose tissues and lipotoxicity may contribute to obesity-mediated hypertension and renal hypoxia, and inflammation-driven neovascularization in the kidneys might be an insufficient compensatory mechanism in this regard [4,5], but whether this is a cause or consequence is still unanswered [6]. Neovascularization is the formation of new capillaries from pre-existing vessels as a functional adaption to a decrease in oxygen supply to the respective tissue, giving rise to increased vessel density or branching degree. Other structural alterations can occur in the vessels in addition to or in combination with neovascularization, such as increased tortuosity, which may lead to ischemic attacks [7]. To improve targeting of obese persons at risk of CKD, it is important to look at renal microcirculation. Quantitative metrics of microvascular structure give insight into the adaptions of microvessel networks and, thereby, the pathological function of the microcirculation, since morphological alterations in vessel architecture are hallmarks of vascular remodeling events. This makes it essential to quantify microvascular changes such as microvessel density and tortuosity [7,8]. However, it is challenging to independently investigate obesity-induced renal vascular injury.

Super-resolution (SR) ultrasound imaging has the potential to visualize the microvasculature in vivo, but the literature on pathological changes in the renal vasculature is limited [9,10,11]. One approach for SR imaging is based on localizing gas-filled intravascular microbubbles (MBs) [12]. By tracking motion-compensated MBs across multiple successive image frames, detailed maps of the vascular network with a spatial resolution below 100 μm can be created [13,14]. The vessels are represented by a number of intraluminal MB tracks/trajectories that are dependent on the intraluminal distribution and number of vessels they pass through [15,16].

This pilot study tested if renal vascular density and tortuosity could be quantified in vivo in obese rats using microbubble-based SR imaging. The study was performed on the obese Zucker rat (OZR). OZR is a metabolic syndrome model with a mutation in the leptin receptor gene that results in obesity within the first 4–5 weeks of age and gives rise to hyperinsulinemia, hyperlipidemia, and hypertension [17,18]. With increasing age, OZRs develop albuminuria (around 14 weeks of age) and focal glomerulosclerosis (around 36 weeks of age), ultimately leading to renal failure manifested by elevated blood urea nitrogen and plasma creatinine [19,20]. In addition to SR imaging, measurements of glomerular filtration rate, urinary albumin-to-creatinine ratio, and renal histopathology were obtained as reference measures.

## 2. Materials and Methods

*Ethical considerations*: The experiments were executed according to protocols approved by the Danish National Animal Experiments Inspectorate and the procedures were performed at the University of Copenhagen, where all local ethical standards were respected. The ethical policy of the university is consistent with that of the National Institutes of Health (NIH). The rats (Envigo, Indianapolis, IN, USA) were obtained at nine weeks of age, fed standard laboratory chow (13198 FORTI) ad libitum, and allowed free access to water. The eight rats were housed in the animal facility at the University of Copenhagen, Department of Experimental Medicine under the responsibility of trained animal caretakers.

*Animal preparation*: The experiments were conducted on two 11-week-old male OZR and two 20-week-old male OZR compared with four healthy age-matched male lean Zucker rats (LZR). One OZR had to be scanned at 17 weeks of age instead of 20 due to health issues. The SR ultrasound scans were performed on anesthetized rats after laparotomy. The rats were anesthetized in an induction chamber with 5% isoflurane delivered in 65% nitrogen and 35% oxygen. The general anesthesia was maintained with an isoflurane concentration of 1.5–2% through a tracheostomy. The rats’ ventilation were controlled by a mechanical ventilator (Ugo Basile, Gemonio, Italy) with a respiration cycle of 69 respirations/min. The blood pressure was measured throughout the experiment by a Statham P23-dB pressure transducer (Gould, Oxnard, CA, USA) via a fluid-filled polyethylene catheter (PE-50) inserted in the left carotid artery. The left jugular vein was catheterized with two polyethylene catheters (PE-10). By the use of a pump (SP210iw syringe pump, WPI, Sarasota, FL, USA) the first catheter provided a continuous infusion of 20 µL/min of the muscle relaxant Nimbex (cisatracurium, 0.85 mg/mL, GlaxoSmithKline, Brentford, UK). The second catheter was used to infuse the contrast agent and a syringe turner ensured a consistent mixture of the MBs by rotating the MB syringe 180° every 10 s [21]. The rats were placed on a heating table to maintain normal body temperature (37 °C). Prior to the scans, plasma and urine samples were collected, as described further below.

**Figure 1:**
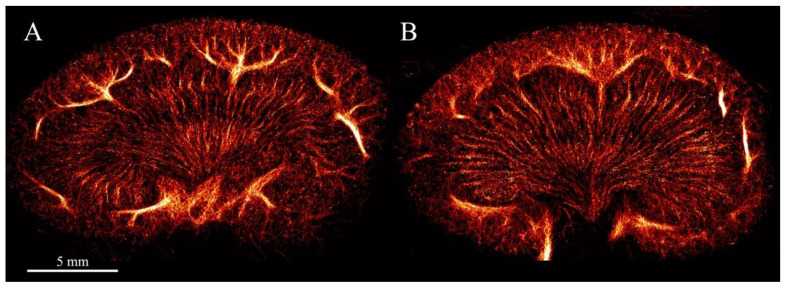
Super-resolution intensity images of the left kidney visualizing the renal vasculature from (**A**) a 20-week-old lean Zucker rat and (**B**) a 20-week-old obese Zucker rat giving an impression of increased density in the cortex with a more chaotic structure of the vessels. The intensity corresponds to the number of tracked microbubbles with a brighter color in the arcuate and segmental vessels compared with the less bright color in the vasa recta of the medulla and smaller vessels in the cortex.

*Ultrasound scan procedure*: To further expose the left kidney and reduce the respiratory motion, a metal retractor was placed under the left side of the diaphragm, pulling slightly cranially. The left kidney was scanned in the coronal view using a modified BK5000 scanner and an X18L5s transducer fixed by a holder (both from BK Medical Aps, Herlev, Denmark). SonoVue (Bracco, Milan, Italy) was used as the contrast agent, diluted in isotonic saline (1:10). The diluted infusion increased the chance of localizing isolated MBs. Each SR ultrasound scan lasted 10 min to make sure that enough MBs were imaged while passing through the renal vascular tree, enabling visualization of the whole vascular bed [15,22]. By using a pulse amplitude modulation sequence, the contrast-enhancing images were obtained with a center frequency (CF) of 6 MHz, a frame rate (FR) of 50 Hz, and a mechanical index (MI) of 0.2. After completing the scans, the rats were euthanized by decapitation while still anesthetized. The left kidney was removed for histology and stored in 4% paraformaldehyde. Tissue motion correction was applied before the tracking of the MBs [13]. The MB tracks were made with the use of a hierarchical Kalman tracker [14]. The SR images were created by inserting the accumulated MB tracks into a 5-µm pixel sized image (Figure 1).

*Blood and urine sampling and analysis*: The blood glucose was measured with the Accu-Chek Aviva system (Roche Diabetes Care, Inc., Indianapolis, IN, USA) on samples from the tail vein prior to laparotomy. A level < 11 mmol/L was determined as normal non-fasting blood glucose. Urine was collected from each rat over a time period of 30 min via a catheter in the left ureter. Urine and plasma creatinine and plasma urea concentrations were measured with Cobas reagents (Roche Diagnostics, Basel, Switzerland) and optimized for small sample volumes. The creatinine measures were used to estimate the GFR as an indicator of renal function. Urine albumin concentrations were measured (ELISA Kit ab108789, Abcam, Cambridge, UK) to assess the glomerular damage. Arterial blood samples were collected before and after the urine collection to estimate the level of plasma creatinine. The mean value of two plasma samples per rat was used. The creatinine clearance was calculated with the following formula:

$$\frac{Urine\;creatinine\;\times \;Urine\;volume}{Plasma\;creatinine\;}$$

Albumin-to-creatinine ratio (ACR) was calculated by dividing albumin concentration in milligrams by creatinine concentration in grams. Samples were stored at −20 °C in the time between experiments and measurements.

*Histopathology*: To evaluate renal tissue damage, the left kidney was stained with Hematoxylin and Eosin (H&E), periodic acid-Schiff (PAS), Sirius Red, and Jones Silver. Sirius Red was used for the detection of tubulointerstitial fibrosis by staining collagen red [23]. PAS staining was used to detect glomerulosclerosis, since it detects potential mesangial expansion and thickening of the glomerular basement membrane (GBM) by staining carbohydrates and carbohydrate rich macromolecules purple [24]. Jones Silver staining shows potential GBM thickening more clearly in a black color compared with PAS staining [24]. The evaluation of the images was performed using light microscopy by an experienced anatomist blinded to the samples.

**Figure 2:**
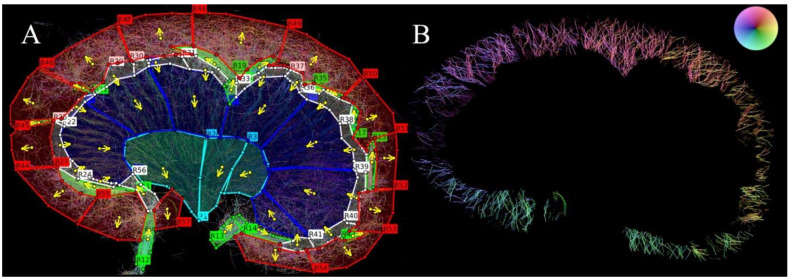
Illustration of the segmentation of a kidney from a 20-week-old obese Zucker rat. (**A**) Red segments define the cortex, the dark blue the outer medulla, the light blue the inner medulla, and the green the larger vessels. The larger vessels were not included in the quantifications of density and tortuosity. The white segments in the sub-cortex were not taken into consideration, since it was difficult to determine whether the tracks in these regions were part of the cortex or the outer medulla. The arrows define the direction of the flow in the regional arteries/arterioles. (**B**) Cortical artery/arteriole tracks after vessel separation. The color of the wheel in the top right corner indicates the flow direction.

*Quantification of renal vascular density and tortuosity*: Anatomical regions (inner medulla (IM), outer medulla (OM), cortex (CO)) were segmented by manually placing regions of interest (ROIs) on the SR images in MATLAB (MathWorks, Natick, MA, USA) (Figure 2A). The IM region was defined by the transition from less dense vasa recta (since it continues unbranched to the tip of the papilla) to a denser vasa recta (since the vessels lie in bundles) in the OM [25]. The vasa recta are, per definition, microvasculature; the ascending vasa recta are venules and descending vasa recta are arterioles. The CO region was defined according to its delimitation by the arcuate arteries and the surface of the kidney [26]. In the cortex, mainly the cortical radial arteries and veins were included, since the current method makes it difficult to distinguish these vessels from the true cortical microvasculature, i.e., the afferent and efferent arterioles, the glomerular and peritubular capillaries, and the cortical venules. The direction of flow was determined in each ROI for separation of arteries and veins, as the arterial flow mainly moves towards the renal surface in the cortex (Figure 2B) and towards the renal papilla in the medulla [27]. When the regions had been segmented, six overall regions per rat were evaluated: three regions that included arteries/arterioles and three regions that included veins/venules. The six different categories were segmented to look at the regions independently and compare regions between OZRs and LZRs separately in young and old rats.

For density and tortuosity estimations, each of the overall regions was automatically divided into 50% overlapping patches with the size of 2 × 2 mm^2^ [28]. In each patch, the local density was calculated as the ratio of non-zero pixels to all of the pixels, as a surrogate for the blood vessel area divided by the total area of that patch. The density was considered a metric between 0 and 1 (1 being maximum density). The tortuosity was calculated as the distance metric: the actual path length from start to end divided by the linear distance between endpoints, which is a metric often used to determine vessel architecture remodeling [28,29].

*Descriptive statistics*: Differences in vessel density were evaluated based on a coefficient. Since the density data are log-transformed the coefficient represents a doubling of the density in OZR compared with LZR if the number is 1, while a negative number represents decreased density in OZR compared with LZR. The descriptive statistics were analyzed using the statistical program R (version 1.4.1106, Vienna, Austria) and GraphPad Prism (version 9.3.1 for Mac, GraphPad Software, San Diego, CA, USA).

## 3. Results

*Biochemical and basic characteristics*: Basic characteristics and biochemical parameters collected and measured at study end are shown in Table 1. Mean arterial blood pressure was slightly higher in the young OZRs and the old OZRs compared with the age-matched LZRs. Blood glucose levels appeared higher in old OZRs, indicating slight hyperglycemia. The albumin excretion rate was higher in OZRs compared with the age-matched LZRs. There was a tendency of decreasing GFR in OZRs compared with the age-matched LZRs and a higher ACR in the old OZRs compared with their age-matched LZRs. The 17-week-old OZR had a very low diuresis (0.0003 mL/min), which did not make it possible to measure urine albumin and creatinine, and consequently, ACR and GFR.

**Figure 3:**
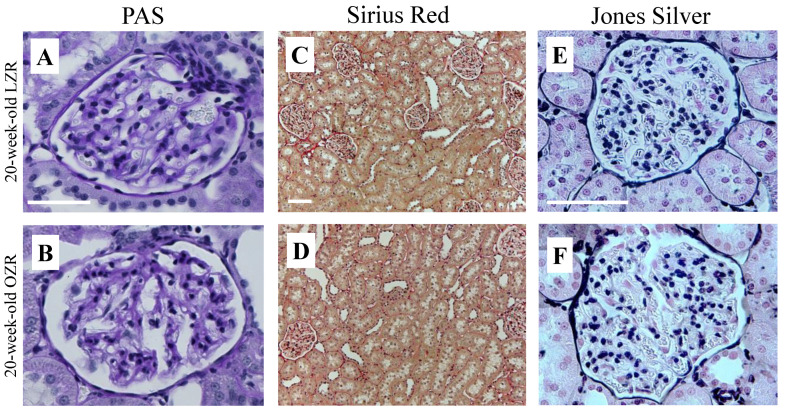
Histology images of kidney tissue from a 20-week-old lean Zucker rat (LZR) and obese Zucker rat (OZR) stained with PAS, Sirius Red, and Jones Silver. Images (**A**,**B**) show glomeruli with no significant mesangial hypertrophy, as the purple-colored carbohydrate would have expanded within the glomerulus. Images (**C**,**D**) primarily show the tubular network without tubulointerstitial fibrosis based on the level of red-stained collagen between the tubules. Images (**E**,**F**) show glomeruli without pathological thickening of the GBM. Scale bar: 50 µm (A + B + E + F), 100 µm (C + D).

*Histopathology evaluation*: The PAS, Sirius Red, and Jones Silver staining (Figure 3) did not reveal glomerulosclerosis by mesangial expansion, GBM thickening, or tubulointerstitial fibrosis in any of the renal tissues, as expected at this age.

*Quantification of the renal vasculature*: Mean number of detected MBs and generated tracks for each of the groups are given in Table 2. A variation in number of MBs between animals was found even though the animals were scanned under similar conditions. For example, the number of MBs and tracks in the CO arteries/arterioles and veins/venules of the young OZRs were only half the amount compared with the amount in young lean rats. Contrarily, the number of MBs detected in the OM arterioles in the old OZRs was notably high, affecting the number of tracks and their quality (a very high MB count will generate more disorganized false tracks).

As illustrated in Table 3, two out of six regions (IM arterioles and venules) showed a denser renal vasculature in young OZRs compared with their age-matched LZRs. The density decrease in CO arteries/arterioles and veins/venules could be related to the difference in detected MBs and, hence, generated tracks: 1225 in LZRs and only 637 in OZRs for the arterioles, and 1632 versus 869 for the veins/venules.

**Figure 4:**
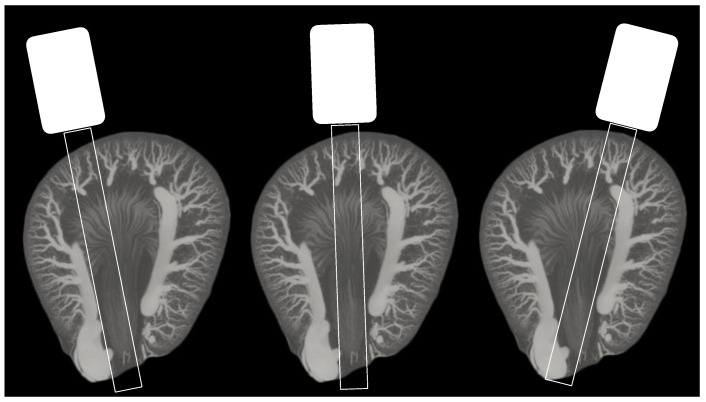
Illustration of three different ultrasound probe positions (coronal slice) on an axial slice maximum intensity projection (50 slices) from an ex vivo µCT of a Sprague Dawley rat kidney filled with intravascular Microfil (from a separate study). The image in the middle demonstrates the optimal position for visualization of inner and outer medulla, as the two other positions can include more of the surrounding tissue, larger vessels, and vasa recta going in the elevational direction.

The old rats showed a denser vasculature in OZR in CO veins/venules and OM venules. The density in CO veins/venules was increased in OZR despite a comparable number of tracks (1974 in OZR and 2128 in LZR). A comparable number of tracks was also present in OM venules: 2222 in OZR and 2074 in LZR. A decrease in old IM arteriole density (1705 in LZR and only 1192 in OZR) was seen despite a similar number of detected MBs. In Figure 4, the possible effect of probe positioning on the medullary vascular density is illustrated and this effect is discussed in the next section.

Comparing tortuosity in the six regions showed no noteworthy differences, as illustrated in Table 4.

## 4. Discussion

This is the first time that the renal vasculature in obese Zucker rats has been quantified by the use of SR ultrasound imaging. The SR images portrayed the renal vascular tree consistent with other imaging methods described in the literature, e.g., magnetic resonance histology [30] and µCT [31]. Changes in the renal microvasculature have been described in other studies using ex vivo µCT. One study quantified the changes in a diabetes type 1 rat model and found a decrease in vessel density [32], and another study described enhanced cortical vascularization of pigs with hypercholesterolemia; both models were investigated at 12 weeks of age [33]. There is an important difference, as the µCTs are performed ex vivo with a scan time of at least 10 h [31], while the in vivo SR ultrasound imaging scan time is only 10 min.

To study the possible progression of renal vascular changes in OZRs with SR imaging, the vascular density and tortuosity were quantified in both 11- and 20-week-old OZRs and age-matched controls. The age of the young rats was selected to have a baseline scan prior to any expected structural changes and the age of the older rats was chosen prior to any glomerulosclerosis found with histology, expected by the age of ~36 weeks [17,20]. Our results showed a difference in vascular density between OZRs and the age-matched LZRs, but the difference varied in all regions. No pathology was detected from the renal tissue stains as expected at this age, but the old OZRs showed signs of renal injury by higher blood pressure, ACR, and lower GFR. Our pilot study was undertaken to investigate the possibilities of quantifying structural alterations in the renal vasculature in vivo. In a larger study, changes in vascular density shown by SR ultrasound imaging could be confirmed by histologically measured microvessel density alterations, as performed by Chen et al. [9], or by using µCT for validation [8].

The vascular density difference varied in all renal regions in the young and old rats. For example, a large difference was found in the cortical arteries/arterioles and veins/venules with a coefficient of −0.68 and −0.57, respectively (Table 3). The young rats were used as a baseline prior to any expected structural alterations, and the substantial difference in density between the OZRs and LZRs was not anticipated. However, the number of detected MBs, and thereby the number of generated tracks, were very different between the young OZRs and LZRs (Table 2). In the 20-week-old group, an increased vascular density was found in the OZRs compared with the age-matched controls in the cortical veins/venules and OM venules corresponding to the observations found using ex vivo µCT [32,33]. However, in this group, the number of tracks also varied in other regions due to the variation in MB detections. The complexity of MB-based super-resolution imaging and the derived vascular representations makes in vivo vessel quantification challenging. In this study, the measured density difference was inevitable, since the density metric is affected by the number of generated tracks. It is important to have a similar number of MBs per frame in all animals and the number of MBs per frame needs to be suitable for the area or organ of interest in order to generate reliable and comparable tracks. However, it is difficult to control number of MBs in vivo, which is a known problem in SR ultrasound imaging [34,35]. To accommodate this challenge, implementation of an MB counter could increase the probability of making the scans homogeneous and reproducible [36]. Alternatively, a feedback system indicating the number of MBs in the contrast-enhancing display could allow more precise adjustments of the MB infusion rate. In addition, the metrics may also be affected by the 2D nature of the SR ultrasound imaging, as slight differences in scan plane can potentially change the quantitative outcome [37]. For example, for the IM, which is thin in the elevational direction, a difference in probe position may affect the number of vasa recta included in the scan plane (Figure 4).

The increased vessel density in the old rats was primarily shown in the vein regions. In a study comparing SR ultrasound imaging with µCT [31], the renal veins in the SR image were visualized with a better correspondence to the renal veins in the µCT than the arteries. This could be due to the fact that the larger renal veins encircle the smaller arteries [31]. It could also be because of the higher blood flow velocity found in the arteries. A very high blood flow velocity complicates tracking of the MBs with a FR of ~50 Hz. We detected a relatively lower number of MBs in the cortical regions compared with the medullary regions, although the cortex receives roughly 90% of the renal blood flow [38,39]. The hierarchical Kalman tracker is challenged by the density and complexity in the cortex, making it difficult to visualize afferent/efferent arterioles and the peritubular capillaries in the cortex [14]. This finding illustrates the difficulty of tracking MBs in vessels with a higher velocity and a more tortuous course, as the vessels in cortex go in and out of plane and might have been filtered away as noisy tracks. MBs are easier to track in the medullary microcirculation with the used scanner settings (CF 6 MHz; FR 50 Hz; MI 0.2), both due to the lower flow velocity and the long straight course of vasa recta in the imaging plane [25]. To meet this limitation, imaging sequences with a higher frame rate would make it possible to capture more MBs and make fewer linking errors, since the tracking is not possible with less than three MBs [14].

The tortuosity is expected to increase in the kidney of an obese rat as a result of lipotoxicity and decrease in kidney function. Our measurements of tortuosity were limited by the 2D technology. Since vessels go in and out of the imaging plane, it is difficult to follow a vessel from its true beginning to its end. This might be one of the reasons, as well as their relatively young age, why no increase in tortuosity when comparing OZR with their age-matched controls was detected in this study.

There are some limitations to the study besides those mentioned above. The most important limitation is the small animal cohort, which of course limits our possibilities to evaluate effects of obesity on the renal vasculature; however, as it is a pilot study, the results have highlighted issues that need attention in future larger-scaled studies. Moreover, even though the ACR (447.29 ± 350.72 and 10,466.52 mg/g in old LZRs and OZRs, respectively) was clearly increased in the old OZRs, which would indicate initial renal pathological changes, we found no histological evidence of manifested CKD, e.g., glomerulosclerosis, nor of tubulointerstitial fibrosis. The rats might not have been old or diseased enough for us to detect significant increases in vessel density in all regions of the kidney; another study used ex vivo µCT to show an increase in the microvascular density of OZRs at week 32 of age [40]. In addition, our rats were fed a regular diet; other studies have found a significant difference in metabolic parameters when the same rat model was fed a high-fat diet for 10 weeks [41]. The high-fat diet accelerates the progression of overt renal disease. Thus, the pathology becomes more pronounced and, as a result, a significant difference between the OZR and LZR may be identified earlier. Going forward, changing the diet and scanning rats at a later stage could be of great importance.

## 5. Conclusions

This study demonstrated the possibility of quantifying the renal vasculature with SR ultrasound imaging. The results showed variation in density due to variation in MB count. When the number of MBs and tracks was comparable, an increased vascular density was found, e.g., CO veins/venules and OM venules in old OZR related to the decrease in GFR and increase in albumin/creatinine ratio. The difference in vascular density was detected prior to changes in stained tissue from the same kidneys. SR imaging has a potential as an additional evaluation method complementing GFR, urinary albumin excretion, and biopsy in determining status of kidney function. SR ultrasound imaging can contribute to visualization of pathological changes in the renal vasculature and could potentially give rise to determination of individuals at risk of developing CKD before it becomes evident with other methods.

## 6. Patents

The tissue motion correction algorithm used in this study is patented by J.A.J. and I.T.

## Figures and Tables

**Table 1:** Basic characteristics and biochemical parameters measured at study end in lean Zucker rats (LZR) and obese Zucker rats (OZR).

	Young		Old	
**Basic characteristics**	LZR	OZR	LZR	OZR
Body weight (g)	329.5 ± 30.4	413 ± 24.0	458 ± 33.9	591.5 ± 6.4
Kidney weight (g)	1.15 *	1.20 *	1.47 ± 0.08	1.51 ± 0.13
KW/BW (%)	0.33	0.28	0.32	0.26
**Biochemical parameters**				
Blood pressure (mmHg)	86.69 ± 10.6	92.60 ± 2.7	89.53 ± 6.9	112.76 ± 5.5
Blood glucose before laparotomy (mmol/L)	-	10.1 ± 1.5	9.05 ± 0.9	11.8 ± 4.9
Plasma creatinine (mg/dL)	0.46 ± 0.4	0.40 ± 0.9	0.53 ± 0.4	0.54 ± 0.5
Plasma urea (mg/dL)	15.86 ± 0.64	20.44 ± 3.25	20.13 ± 1.15	22.42 ± 3.10
Urinary creatinine (mg/dL)	86.00 ± 52.61	19.50 ± 9.97	40.71 ± 12.47	10.39 **
Urinary albumin (mg/dL)	6.67 ± 6.11	6.83 ± 0.86	20.97 ± 22.04	102.62 ± 36.70
Creatinine excretion rate (mg/min)	0.007	0.004	0.006	0.002 **
Albumin excretion rate (mg/min)	0.0005	0.001	0.003	0.02
Albumin/creatinine ratio (mg/g)	65.15 ± 35.78	413.35 ± 171.87	447.29 ± 350.72	10,466.52 **
GFR/Creatinine clearance (ml/min)	0.63 ± 0.06	0.41 ± 0.01	0.57 ± 0.31	0.43 **
Diuresis (µl/min)	7.57 ± 2.71	19.53 ± 5.33	15.43 ± 5.73	22.63 ± 15.58

Values are means ± SD, n = 2. * One weight. ** One rat due to the lack of urine production from the 17-week-old OZR.

**Table 2:** Number of tracks and microbubbles (MBs) after filtering in the three different areas separated into arteries (A) and veins (V) in the kidney of obese Zucker rats (OZRs) and lean Zucker rats (LZRs), 11- and 20-weeks-old, respectively.

Rat age	**Young**											
Rat type	LZR						OZR					
Area	**IM**		**OM**		**CO**		**IM**		**OM**		**CO**	
Vesselkind	**A**	**V**	**A**	**V**	**A**	**V**	**A**	**V**	**A**	**V**	**A**	**V**
No. of tracks	1036	661	2173	1565	1225	1632	890	627	1623	1265	637	869
No. of MBs	32,341	29,091	59,117	60,303	18,43	22,904	34,282	25,838	49,267	42,671	9524	12,612
Rat age	**Old**											
Rat type	LZR						OZR					
Area	**IM**		**OM**		**CO**		**IM**		**OM**		**CO**	
Vesselkind	**A**	**V**	**A**	**V**	**A**	**V**	**A**	**V**	**A**	**V**	**A**	**V**
No. of tracks	1705	865	3021	2024	1400	2128	1192	658	4201	2222	1592	1974
No. of MBs	45,215	32,365	76,814	64,762	22,565	31,639	42,857	28,929	140,094	86,223	29,232	32,476

The numbers represent the average number of tracks and MBs from two rats in each group. IM—inner medulla, OM—outer medulla, CO—cortex.

**Table 3:** Comparison of renal vascular density between lean Zucker rats (LZR) and obese Zucker rats (OZR) at the age of 11 and 20 weeks.

Vascular Density	Young	Old
	OZR vs. LZR	OZR vs. LZR
CO arteries/arterioles	−0.68 [−0.81;−0.55]	−0.04 [−0.17;0.09]
CO veins/venules	−0.57 [−0.69;−0.45]	0.23 [0.12;0.34]
OM arterioles	−0.16 [−0.25;−0.06]	0.02 [−0.07;0.12]
OM venules	−0.06 [−0.21;0.09]	0.42 [0.29;0.55]
IM arterioles	0.31 [0.13;0.50]	−0.20 [−0.31;−0.08]
IM venules	0.20 [0.03;0.38]	0.01 [−0.12;0.15]

The coefficient defines how far from the reference (LZR) the OZR data are. The coefficient represents a doubling of the density in OZR compared with LZR if the number is 1, while a negative number demonstrates lower density in OZR compared with LZR. n = 2. [CI 95].

**Table 4:** Renal vascular tortuosity in lean Zucker rats (LZR) and obese Zucker rats (OZR) at the age of 11 and 20 weeks.

Vascular Tortuosity	Young		Old	
	LZR	OZR	LZR	OZR
CO arteries/arterioles	1.12	1.12	1.13	1.13
CO veins/venules	1.12	1.12	1.13	1.13
OM arterioles	1.12	1.11	1.12	1.11
OM venules	1.13	1.13	1.13	1.14
IM arterioles	1.12	1.11	1.12	1.12
IM venules	1.13	1.13	1.13	1.13

The numbers represent the median tortuosity, n = 2.

## Data Availability

Analysis algorithms and processed data can be accessed upon request.

## References

[1] World Health Organization (2021). Obesity and Overweight. https://www.who.int/news-room/fact-sheets/detail/obesity-and-overweight.

[2] Wang Y., Chen X., Song Y., Caballero B., Cheskin L.J. (2008). Association between Obesity and Kidney Disease: A Systematic Review and Meta-Analysis. Kidney Int..

[3] Cao X., Zhou J., Yuan H., Wu L., Chen Z. (2015). Chronic Kidney Disease among Overweight and Obesity with and without Metabolic Syndrome in an Urban Chinese Cohort Epidemiology and Health Outcomes. BMC Nephrol..

[4] Hall M.E., do Carmo J.M., da Silva A.A., Juncos L.A., Wang Z., Hall J.E. (2014). Obesity, Hypertension, and Chronic Kidney Disease. Int. J. Nephrol. Renovasc. Dis..

[5] Chade A.R., Hall J.E. (2016). Role of the Renal Microcirculation in Progression of Chronic Kidney Injury in Obesity. Am. J. Nephrol..

[6] Costa C., Incio J., Soares R. (2007). Angiogenesis and Chronic Inflammation: Cause or Consequence?. Angiogenesis.

[7] Han H.C. (2012). Twisted Blood Vessels: Symptoms, Etiology and Biomechanical Mechanisms. J. Vasc. Res..

[8] Ehling J., Bábícková J., Gremse F., Klinkhammer B.M., Baetke S., Knuechel R., Kiessling F., Floege J., Lammers T., Boor P. (2016). Quantitative Micro-Computed Tomography Imaging of Vascular Dysfunction in Progressive Kidney Diseases. J. Am. Soc. Nephrol..

[9] Chen Q., Yu J., Rush B.M., Stocker S.D., Tan R.J., Kim K. (2020). Ultrasound Super-Resolution Imaging Provides a Noninvasive Assessment of Renal Microvasculature Changes during Mouse Acute Kidney Injury. Kidney Int..

[10] Qiu L., Zhang J., Yang Y., Zhang H., Lee F., He Q., Huang C., Huang L., Qian L., Luo J. (2022). In Vivo Assessment of Hypertensive Nephrosclerosis Using Ultrasound Localization Microscopy. Med. Phys..

[11] Andersen S.B., Taghavi I., Hoyos C.A.V., Søgaard S.B., Gran F., Lönn L., Hansen K.L., Jensen J.A., Nielsen M.B., Sørensen C.M. (2020). Super-Resolution Imaging with Ultrasound for Visualization of the Renal Microvasculature in Rats Before and After Renal Ischemia: A Pilot Study. Diagnostics.

[12] Christensen-Jeffries K., Couture O., Dayton P.A., Eldar Y.C., Hynynen K., Kiessling F., O’Reilly M., Pinton G.F., Schmitz G., Tang M.X. (2020). Super-Resolution Ultrasound Imaging. Ultrasound Med. Biol..

[13] Taghavi I., Andersen S.B., Hoyos C.A.V., Nielsen M.B., Sorensen C.M., Jensen J.A. (2021). In Vivo Motion Correction in Super Resolution Imaging of Rat Kidneys. IEEE Trans. Ultrason. Ferroelectr. Freq. Control.

[14] Taghavi I., Andersen S.B., Hoyos C.A.V., Schou M., Gran F., Hansen K.L., Nielsen M.B., Sørensen C.M., Stuart M.B., Jensen J.A. (2022). Ultrasound Super-Resolution Imaging with a Hierarchical Kalman Tracker. Ultrasonics.

[15] Hingot V., Errico C., Heiles B., Rahal L., Tanter M., Couture O. (2019). Microvascular Flow Dictates the Compromise between Spatial Resolution and Acquisition Time in Ultrasound Localization Microscopy. Sci. Rep..

[16] Christensen-Jeffries K., Brown J., Harput S., Zhang G., Zhu J., Tang M.X., Dunsby C., Eckersley R.J. (2019). Poisson Statistical Model of Ultrasound Super-Resolution Imaging Acquisition Time. IEEE Trans. Ultrason. Ferroelectr. Freq. Control.

[17] Schmitz P.G., O’Donnell M.P., Kasiske B.L., Katz S.A., Keane W.F. (1992). Renal Injury in Obese Zucker Rats: Glomerular Hemodynamic Alterations and Effects of Enalapril. Am. J. Physiol. Ren. Fluid Electrolyte Physiol..

[18] Zucker L.M. (1965). Hereditary Obesity in the Rat Associated with Hyperlipidemia. Ann. N. Y. Acad. Sci..

[19] Gades M.D., Van Goor H., Kaysen G.A., Johnson P.R., Horwitz B.A., Stern J.S. (1999). Brief Periods of Hyperphagia Cause Renal Injury in the Obese Zucker Rat. Kidney Int..

[20] Kasiske B.L., O’Donnell M.P., Cleary M.P., Keane W.F. (1988). Treatment of Hyperlipidemia Reduces Glomerular Injury in Obese Zucker Rats. Kidney Int..

[21] Lohmaier S., Ghanem A., Veltmann C., Sommer T., Bruce M., Tiemann K. (2004). In Vitro and in Vivo Studies on Continuous Echo-Contrast Application Strategies Using SonoVue in a Newly Developed Rotating Pump Setup. Ultrasound Med. Biol..

[22] Taghavi I., Andersen S.B., Hoyos C.A.V., Schou M., Øygard S.H., Gran F., Hansen K.L., Sørensen C.M., Bachmann Nielsen M., Stuart M.B. Tracking Performance in Ultrasound Super-Resolution Imaging. Proceedings of the 2020 IEEE International Ultrasonics Symposium (IUS).

[23] Farris A.B., Alpers C.E. (2014). What Is the Best Way to Measure Renal Fibrosis¿: A Pathologist’s Perspective. Kidney Int. Suppl..

[24] Amann K., Haas C.S. (2006). What You Should Know about the Work-up of a Renal Biopsy. Nephrol. Dial. Transplant..

[25] Zimmerhackl B., Robertson C.R., Jamison R.L. (1985). The Microcirculation of the Renal Medulla. An Off. J. Am. Heart. Assoc. Br. Rev..

[26] Moffat D.B., Fourman J. (1963). The Vascular Pattern of the Rat Kidney. J. Anat..

[27] Dalal R., Bruss Z.S., Sehdev J.S. (2021). Physiology, Renal Blood Flow and Filtration. StatPearls.

[28] Taghavi I., Andersen S.B., Sogaard S.B., Nielsen M.B., Sorensen C.M., Stuart M.B., Jensen J.A. Automatic Classification of Arterial and Venous Flow in Super-Resolution Ultrasound Images of Rat Kidneys. Proceedings of the 2021 IEEE International Ultrasonics Symposium (IUS).

[29] Lowerison M.R., Huang C., Lucien F., Chen S., Song P. (2020). Ultrasound Localization Microscopy of Renal Tumor Xenografts in Chicken Embryo Is Correlated to Hypoxia. Sci. Rep..

[30] Xie L., Cianciolo R.E., Hulette B., Lee H.W., Qi Y., Cofer G., Johnson G.A. (2012). Magnetic Resonance Histology of Age-Related Nephropathy in the Sprague Dawley Rat. Toxicol. Pathol..

[31] Andersen S.B., Taghavi I., Kjer H.M., Søgaard S.B., Gundlach C., Dahl V.A., Nielsen M.B., Dahl A.B., Jensen J.A., Sørensen C.M. (2021). Evaluation of 2D Super-Resolution Ultrasound Imaging of the Rat Renal Vasculature Using Ex Vivo Micro-Computed Tomography. Sci. Rep..

[32] Maric-Bilkan C., Flynn E.R., Chade A.R. (2012). Microvascular Disease Precedes the Decline in Renal Function in the Streptozotocin-Induced Diabetic Rat. Am. J. Physiol. Physiol..

[33] Bentley M.D., Rodriguez-Porcel M., Lerman A., Hershman Sarafov M., Romero J.C., Pelaez L.I., Grande J.P., Ritman E.L., Lerman L.O. (2002). Enhanced Renal Cortical Vascularization in Experimental Hypercholesterolemia. Kidney Int..

[34] Dencks S., Piepenbrock M., Opacic T., Krauspe B., Stickeler E., Kiessling F., Schmitz G. (2019). Clinical Pilot Application of Super-Resolution US Imaging in Breast Cancer. IEEE Trans. Ultrason. Ferroelectr. Freq. Control.

[35] Huang C., Zhang W., Gong P., Lok U.W., Tang S., Yin T., Zhang X., Zhu L., Sang M., Song P. (2021). Super-Resolution Ultrasound Localization Microscopy Based on a High Frame-Rate Clinical Ultrasound Scanner: An in-Human Feasibility Study. Phys. Med. Biol..

[36] Ghosh D., Peng J., Brown K., Sirsi S., Mineo C., Shaul P.W., Hoyt K. (2019). Super-Resolution Ultrasound Imaging of Skeletal Muscle Microvascular Dysfunction in an Animal Model of Type 2 Diabetes. J. Ultrasound Med..

[37] Foiret J., Zhang H., Ilovitsh T., Mahakian L., Tam S., Ferrara K.W. (2017). Ultrasound Localization Microscopy to Image and Assess Microvasculature in a Rat Kidney. Sci. Rep..

[38] Duke J. (2011). Renal Function and Anesthesia. Anesthesia Secrets.

[39] Damkjær M., Vafaee M., Møller M.L., Braad P.E., Petersen H., Høilund-Carlsen P.F., Bie P. (2010). Renal Cortical and Medullary Blood Flow Responses to Altered NO Availability in Humans. Am. J. Physiol. Regul. Integr. Comp. Physiol..

[40] Iliescu R., Chade A.R. (2010). Progressive Renal Vascular Proliferation and Injury in Obese Zucker Rats. Microcirculation.

[41] Ebenezer P.J., Mariappan N., Elks C.M., Haque M., Soltani Z., Reisin E., Francis J. (2009). Effects of Pyrrolidine Dithiocarbamate on High-Fat Diet-Induced Metabolic and Renal Alterations in Rats. Life Sci..

